# Editorial: Lectins from plants, algae, fungi, bacteria and animal therapeutic tools for SARS-CoV-2 and other pathogenic enveloped viruses, in a “one-health” perspective

**DOI:** 10.3389/fcimb.2023.1139691

**Published:** 2023-01-23

**Authors:** Els J. M. Van Damme, Pierre Rougé

**Affiliations:** ^1^ Biotechnology, Ghent University, Ghent, Belgium; ^2^ Pharmadev, Université Toulouse III Paul Sabatier, Toulouse, France

**Keywords:** enveloped virus, lectin, glycan, carbohydrate recognition, carbohydrate-binding agent

Lectins or carbohydrate-binding agents (CBAs) from diverse sources including plants, algae, fungi, cyanobacteria and animals, can recognize the glycan chains decorating pathogenic enveloped viruses, and consequently offer an interesting alternative to antibodies and antiviral drugs for combating viral infections. Most of the pathogenic enveloped viruses are highly *N*-glycosylated and exhibit surface glycoproteins containing different types of *N*-glycans, including complex-type glycans, high-mannose- or oligomannoside-type glycans, and hybrid-type glycans, representing potential targets for lectins with different carbohydrate-binding specificities (Barre et al., 2022). In this regard, the perspective paper from Maier, demonstrated that natural cyanovirin-N (CV-N), the mannose-specific lectin from the cyanobacterium *Nostoc ellipsosporum*, specifically recognizes *N*-glycans of the high-mannose type occurring on the surface of hemagglutinin from influenza virus, gp120 from human immunodeficiency virus 1 (HIV-1) and spike protein S from SARS-CoV and SARS-CoV-2. In addition, an engineered recombinant cyanovirin-N (CV-N) domain-swapped dimer with enhanced binding capacities towards SARS-CoV-2 showed increased binding affinity to the highly *N*-glycosylated spike protein and hemagglutinin. These engineered CV-N dimers with enhanced binding capacities could be used to improve the neutralization ability of the lectin towards pathogenic enveloped viruses by reducing both the internalization and the spreading of the viruses.

However, the antiviral activity of lectins is not limited to hampering or preventing the recognition and subsequent anchorage of the viral particles to the host cells. The Original Research article of Vanhulle et al., brings interesting insights into the antiviral activity of the stinging nettle (*Urtica dioica*) lectin UDA, a small monomeric lectin with a specificity for GlcNAc, towards the SARS-CoV-2 Wuhan-Hu-1 strain and other SARS-CoV-2 variants of concern (VOCs). At nanomolar concentration, UDA prevented the viral replication of the SARS-CoV-2 Wuhan in Vero E6 cells but also the replication of SARS-CoV-2 VOCs Alpha, Beta and Gamma. Similarly, UDA inhibited the viral replication of VOCs Delta and Omicron in U87.ACE2^+^ cells. These results demonstrate that the antiviral activity of UDA relies primarily on the recognition of the glycan shield, which is apparently similarly *N*- and *O*-glycosylated in the SARS-CoV-2 WT and the VOCs. However, in addition to interfere with the anchorage and subsequent entry of SARS-CoV-2 WT and VOCs into the host cells, UDA also appears as a potent fusion inhibitor for the betacoronaviruses, as shown from cell-cell fusion experiments. Finally, the S1 *N-*terminal part of the SARS-CoV-2 spike protein was identified as the main target for UDA since SARS-CoV-2 mutants exhibiting *N*-glycosylation deletions in the S2 *C*-terminal portion of the spike protein, remained sensitive to the antiviral activity of UDA.

Another well-known algal lectin with a specificity for mannose, griffithsin (GRFT), was investigated for its antiviral properties *in vitro* and *in vivo* towards the Hantaan virus (HTNV) responsible for hemorrhagic fever in East Asia, in the Original Research article from Zhao et al.. *In vitro* experiments using recombinant vesicular stomatitis virus (rVSV) bearing HTNV glycoproteins revealed that GRFT inhibited the entry of rVSV-HTNV in cultured host cells, by binding to the HTNV *N*-glycans and preventing the virus from interacting with the cellular receptors and subsequent entry in the cells. In addition, a reduction in mouse mortality of suckling mice infected by HTNV, was obtained after GRFT treatment. These results suggest that GRFT could be a promising therapeutic agent for preventing and treating the HTNV infection.

Finally, the Original Research article from Nabeta et al., addresses the antifungal activity of an engineered griffithsin (Q-GRFT) with an improved stability, for inhibiting *Candida* infection in murine models of vaginal candidiasis. *In vivo* experiments showed that Q-GRFT treatment of infected mice significantly lowered the fungal burden, compared with the infected non-treated mice. In addition, a repeated Q-GRFT treatment over 7 days enhanced the clearance of vaginal candidiasis in previously infected mice. A histological control of vaginal tissues confirmed the efficacy of the Q-GRFT treatment on infected mice. These *in vivo* results demonstrate that griffithsin can inhibit *Candida* growth, and *in vitro* experiments suggest that the inhibitory effect is independent of the inflammatory immune response caused by candidiasis. Although the binding of griffithsin to the *Candida* cells is based on the recognition of mannose-containing glycans present on the cell surface, this article addresses the anti-fungal activity of griffithsin rather than its antiviral activity against enveloped viruses.

Although the articles of the Research Topic bring new interesting information on the antiviral properties of cyanobacterial, algal and plant lectins, it is regrettable that animal lectins and in particular, C-lectins, have not been considered as CBAs for enveloped viruses in this topic. In this respect, some of the molecules forming the humoral arm of innate immunity recognize various pathogenic enveloped viruses including influenza A virus, HIV-1, *Herpes simplex* virus and hepatitis C virus (Holmskov et al., 2003). Accordingly, recent results showed that pentraxin 3 (PTX3) and mannose-binding lectin (MBL) readily anchor to the nucleocapsid and spike proteins of SARS-CoV-2 and its Omicron VOC, respectively. At nanomolar concentration, the collectin MBL binds to the SARS-CoV-2 spike protein in a glycan-dependent manner and inhibits the infection of primary respiratory cells by SARS-CoV-2. Moreover, the bound MBL triggers the activation of the lectin pathway of complement activation (Stravalaci et al., 2022).

## Author contributions

All authors listed have made a substantial, direct, and intellectual contribution to the work and approved it for publication.
